# Correction: Optimization of CFTR gating through the evolution of its extracellular loops

**DOI:** 10.1085/jgp.20221326403272024c

**Published:** 2024-04-04

**Authors:** Márton A. Simon, László Csanády

Vol. 155, No. 4 | https://doi.org/10.1085/jgp.202213264 | February 01, 2023

The authors regret that, in the abstract of their originally published article, zS120 was inadvertently written instead of zN120. The corrected abstract appears below with the correction in bold. This error appears in print and PDFs downloaded before March 28, 2024.

CFTR chloride channel mutations cause the lethal and incurable disease cystic fibrosis (CF). CFTR is activated by phosphorylation, and phosphorylated channels exhibit “bursting” behavior—“bursts” of openings separated by short “flickery” closures and flanked by long “interburst” closures—driven by ATP binding/hydrolysis at two nucleotide-binding domains. The human channel (hCFTR) and the distant zebrafish ortholog (zCFTR) display differences both in their gating properties and structures. In phosphorylated ATP-bound hCFTR, the hR117 side chain, conserved across evolution, forms an H-bond that stabilizes the open state. Lack of that bond in the hR117H mutant causes CF. In the phosphorylated ATP-bound zCFTR structure that H-bond is not observable. Here, we show that the zR118H mutation does not affect the function of zCFTR. Instead, we identify an H-bond between the zS109 and **zN120** side chains of phosphorylated ATP-bound, but not of unphosphorylated apo-, zCFTR. We investigate the role of that interaction using thermodynamic mutant cycles built on gating parameters determined in inside-out patch clamp recordings. We find that zS109 indeed forms an H-bond with zN120 in the flickery closed state, but not in the open or interburst closed states. Although in hCFTR an isoleucine (hI119) replaces the asparagine, mutation hS108A produces a strong hR117H-like phenotype. Since the effects of the latter two mutations are not additive, we conclude that in hCFTR these two positions interact, and the hS108-hR117 and hR117-hE1124 H-bonds cooperate to stabilize the open state. These findings highlight an example of how the gating mechanism was optimized during CFTR molecular evolution.

